# Ammonium Release and Adsorption Characters of Polyurethane–Biochar Crosslinked Material as an Additive Filler in Stormwater Treatment

**DOI:** 10.3390/polym13101557

**Published:** 2021-05-13

**Authors:** Yuan Wang, Yike Meng, Chuanyue Wang, Bao Wang

**Affiliations:** 1 College of Civil and Transportation Engineering, Hohai University, Nanjing 210098, China; 19860001@hhu.edu.cn; 2 College of Water Conservancy and Hydropower Engineering, Hohai University, Nanjing 210098, China; 3 China Construction Infrastructure Co., Ltd., Beijing 100029, China; wbqymx@163.com

**Keywords:** polyurethane–biochar crosslinked material, bioretention system, ammonia nitrogen leaching, ammonium adsorption, stormwater treatment

## Abstract

The additive fillers in bioretention facilities play a leading role in stormwater treatment to purify polluted runoff. At present, many traditional materials could not meet the requirements at the same time, including low ammonium leaching quantities, high water storage volume and strong ammonium adsorption. This study investigated a polymer material, polyurethane–biochar crosslinked material (PCB), to evaluate the feasibility of using it as an additive filler in stormwater treatment compared with its raw material hardwood biochar (HB), and two traditional fillers. Successive leaching and ammonium isothermal adsorption experiments were conducted in deionized water and artificial stormwater. PCB leached 4.98–5.31 μmol/g NH_4_-N, less than the leaching quantities of compost, the traditional filler. After polyurethane modification, ammonium adsorption of PCB was improved: at a typical ammonium concentration of 2 mg/L in stormwater, PCB could adsorb 43.6 mg/kg ammonium versus 34.6 mg/kg for HB. With the addition of PCB in sand column, the ammonium adsorption improved from 31.34 to 84.72%. To improve the performance of bioretention facilities, PCB is recommended to be added into filter layers in stormwater treatment, taking advantage of its high cation exchange capacity and spongy internal structure to minimize overland flooding and enhance removal of ammonium from stormwater.

## 1. Introduction

Currently, the rapid development of urbanization not only brings convenience, but also brings ecological problems that break the balance of the water resource cycle [1]. The balance of the water resource cycle is fundamental to sustain human development, as it realizes the migration and transformation of water, energy and geochemical substances in the earth system [2]. Human activities in urban areas aggravates the non-point source pollution of water resources, especially ammonium pollution [3]. Ammonium is toxic for aquatic systems, and might lead to the accumulation of nitrite [4]. What is worse, the water cycle intensifies the diffusion of pollutants [5]. Bioretention systems are designed to address the imbalance of the water cycle and stormwater runoff pollution [6]. They can remove dissolved pollutants from stormwater runoff and reduce the peak volume of runoff [7].

A bioretention system, from top to bottom, generally consists of a vegetation layer, a filtration layer, a transition layer and a drainage layer [8]. To achieve the ideal performance in stormwater treatment, the fillers in the filtration layer are crucial. The traditional fillers in a filtration layer are a mixture of 30–60% sand, 20–30% soil and 20–40% compost by volume [9]. Through laboratory and field tests, scholars have found that the traditional filtration layer of bioretention facilities has certain but unstable purifying effects on water quality: high removal efficiency of oil [10], heavy metals [11], pathogenic bacteria indicator species [12], et cetera, but an unstable effect on the removal of nutrients [13]. This is mainly due to the leaching of ammonium and phosphorus from the compost [14].

Efforts have been made to improve the performance of the filtration layer in bioretention systems: Some scholars use mineral materials as additive fillers, including volcanic rocks [15], vermiculite, ceramicite, et cetera. Although they have reduced ammonium leaching, their adsorption capacity to water and pollutants is far less than that of biological waste materials (e.g., alkaline solid wastes [16] and coconut coir [17]). Another additive filler, biochar, has been proposed in stormwater treatment for its cleanness [18] and nutrient adsorption capacity [19]. Yet along with it, the powder size of the biochar results in relatively lower saturated moisture content (125–152%) [20] and water retention capacity [21], which could not cope with flood peaks during storms, leading to outflow and runoff pollution. The current fillers in a filtration layer cannot meet the needs of stormwater treatment, which are a high hydraulic conductivity to minimize overland flooding and a high storage volume to reduce peak flow and enhance removal of many pollutants from stormwater [22].

Polyurethane composite materials could probably solve this challenge. Polyurethane materials have good hydrophilicity and water retention capacity [23], which will help to achieve the aim of reducing runoff peak and volume in stormwater treatment, if they were applied in bioretention facilities. From the perspective of water quality enhancement, polyurethane sponge modified soil improved nitrogen removal rates and lifespan when treating septic tank effluent, because the polyurethane modification can improve the ratio of pores and support more biomat in the fortified soil [24]. As for nitrogen leaching, the releasing quantities and rates become slower when fertilizers are coated by the polyurethane [25]. In addition, our recently published article has also proved that polyurethane–biochar crosslinked material (PCB) is a new polymer material which has high water retention capacity, low phosphorus leaching quantities, as well as high phosphorus adsorption capacity [26]. The network of polyurethane in PCB restrained the release of phosphorus from interpenetrated biochar, and promoted the phosphate removal rate form the runoff. While demonstrating positive results, it is still unclear if whether the addition of polyurethane composite material in bioretention facilities will help to improve the purification efficiency of ammonium in stormwater treatment, and how polyurethane cooperates with its partner material.

This study is a continuation of former work to evaluate the feasibility of using polyurethane–biochar crosslinked material (PCB) as an additive filler to enhance ammonium adsorption in stormwater treatment, and the possibility to reduce NH_4_-N release. For comparison, hardwood biochar (the raw material of PCB) and two traditional fillers (volcanic stone and compost) have also been investigated.

## 2. Materials and Methods

### 2.1. Additive Fillers Preparation

Polyurethane–biochar crosslinked material (PCB) was synthesized with a simple one-shoot method, where glycol, deionized water and hardwood biochar were mixed with methylene diphenyl diisocyanate (MDI). For the formulations and detailed polymerization process of PCB, readers can refer to the published article [26].

The research chose three types of traditional additive fillers in stormwater treatment: compost (CO), volcanic stone (VS) and hardwood biochar (HB), to assess the feasibility of PCB in bioretention systems and to discover its working mechanism. In consideration of the practical application and consistent research scale of these fillers, the cured PCB was cut to a particle size of 1–2 mm.

### 2.2. The Physicochemical and Thermal Characterizations Tests

The physicochemical characterizations indicators of additive fillers include natural bulk density (*ρ*), the saturated moisture content (*ω**_sat_***), particle size, specific gravity, pore ratio (*e*), permeability coefficient (*K*), specific surface area (BET), pH, cation exchange capacity (CEC), total nitrogen content (TN), scanning electron microscopy (SEM) and energy dispersive spectroscopy (EDS). The methods of testing the physicochemical characterizations of additive fillers were consistent with the published article [26]. Additionally, TN of additive fillers was measured by combustion using a Multi N/C2100 Elemental Analyzer (Jena, Germany).

The thermal behavior of PCB was characterized by thermogravimetric analysis (TGA) and differential scanning calorimetry (DSC). TGA was performed using a thermal gravimetric analyzer device (TGA2, Mettler Toledo, Switzerland) in a nitrogen atmosphere at a temperature range of 20–700 °C with a heating rate of 20 °C/min. DSC was tested using a differential scanning calorimetry device (DSC3, Mettler Toledo, Switzerland) in a nitrogen atmosphere. The measurements were performed on 5.67 mg of PCB at a temperature increasing to 200 °C at a heating rate of 200 °C/min, holding for 1 min and quenching to −30 °C, and heated again to 200 °C at a heating rate of 20 °C/min.

### 2.3. Leaching Experiments

The main reason for the unstable efficiency of ammonium removal in stormwater treatment is the leaching of ammonia nitrogen (NH_4_-N) from additive filler materials. To evaluate NH_4_-N leaching quantities, materials were continuously rinsed with deionized water (DW) or artificial stormwater (AS), and the release characteristics of NH_4_-N were analyzed. AS was a mixture solution of 120 mg/L CaCl_2_ and 3 mg/L Na_2_HPO_4_ at pH 7.0, referring to the recognized makeup of synthetic urban runoff [10]. In order to reduce the influence of other factors, motor oil, heavy metals and nitrogen were not added to AS. Furthermore, 5 g of materials, dried at 60 °C for 48 h, were added to a conical flask containing 100 mL of DW or AS. At 20 ± 2 °C, they were oscillated at a frequency of 150 rpm for 24 h. After settlement for 30 min, supernatants were aspirated into centrifuge tubes, centrifuged at 5000 rpm (relative centrifugal force: 4390× *g*) for 20 min. The supernatants were filtered with 0.45 μm filters, and analyzed for NH_4_-N and water conductivity. Another 100 mL of DW or AS was added into the conical flasks, and the leaching−settling−centrifuging steps repeated, until the conductivities of supernatants were constant compared to the last round. Conical flasks containing only DW or AS without materials were set as the control groups. Two sets of tests were repeated for each material. After leaching experiments, the DW rinsed materials were put into a desiccator for later tests.

The San^++^ Continuous Flow Analyzer (Skalar, Dutch) was used for the testing of NH_4_-N. In an alkaline environment, NH_4_-N in the samples reacts with hypochlorite to form chloramine. Chloramine reacts with salicylic acid (C_7_H_6_O_3_) to form blue-green compounds in the presence of potassium nitroferricyanide (C_5_H_4_FeN_6_Na_2_O_3_) at 60 °C. Colorimetric analysis was carried out at a 660 nm wavelength for the detection of NH_4_-N with a detection limit of 0.004 mg/L.

### 2.4. Adsorption Experiments

As additive fillers in stormwater runoff treatment, the materials need to have certain adsorption capacities of the ammonium in stormwater treatment. In order to evaluate the ammonium adsorption capacity of the materials, the adsorption experiments were conducted on DW rinsed materials in different concentrations of ammonium solutions. The standard solution of 100 mg/L NH_4_Cl was diluted to 0.5, 1, 2, 5, 7 and 10 mg/L with DW, respectively. Furthermore, 0.2 g of DW rinsed materials were put into a 50 mL conical flask, and 10 mL of the above concentration solution was added into respective flasks. The conical flasks were oscillated for 24 h at 150 rpm at 20 ± 2 °C. The method of extracting supernatants and detecting ammonium were the same as the leaching tests. The experiments were repeated in 2 groups for each material. Additional conical flasks with only ammonium solutions were used as controls.

In order to explore the ammonium adsorption properties and capacities of additive fillers, Langmuir and Freundlich models [27] were used to fit the adsorption equilibrium quantities *q_e_* (mg/kg) of ammonium after 24 h with the Equations (1) and (2):

(1) $$q_{e}=K_{F}Ce^{1/n},$$

(2) $$q_{e}=q_{\max}\frac{K_{L}C_{e}}{1+K_{L}C_{e}}$$

where, *C_e_* are the concentrations (mg/L) of ammonium in the solution after the adsorption tests; *K_F_* is the volume-affinity parameter (L/mg) of the Freundlich model; *n* is the Freundlich characteristic constant; *q*_max_ is the maximum adsorption capacity (mg/kg); and *K_L_* is the affinitive parameter of the Langmuir model (L/mg). Dimensionless coefficient *R_L_* is used to determine whether adsorption occurs easily:

(3) $$R_{L}=\frac{1}{1+K_{L}C_{0}}$$

When 0 < *R_L_* < 1, adsorption occurs easily; when *R_L_* > 1, adsorption does not occure easily; when *R_L_* = 0, the adsorption process is reversible; and when *R_L_* = 1, it is linear adsorption.

### 2.5. Column Experiments

**Figure 1:**
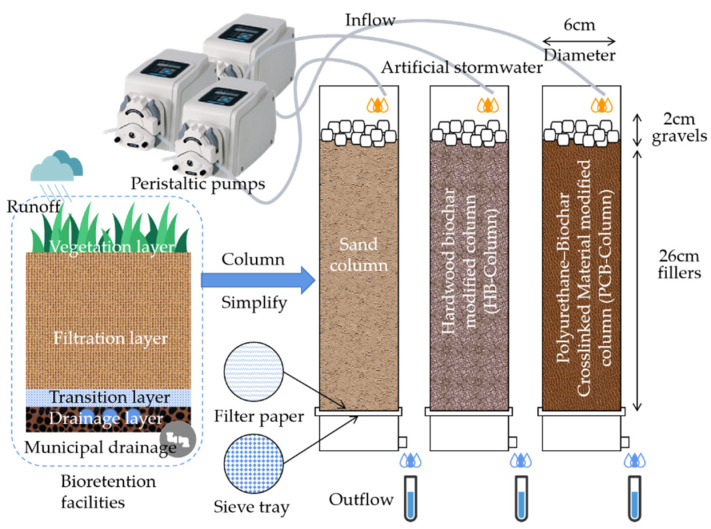
Schematic diagram of column experiments.

In order to evaluate the feasibility of using PCB as an additive filler to enhance ammonium adsorption in stormwater treatment, column experiments were conducted as simplified bioretention facilities, as shown in Figure 1. Three PVC columns, which are denoted as PCB-Column, HB-Column and Sand-Column, were set to evaluate the performance of the additive fillers. In the PCB-Column, the river sand (washed by DW and dried) and PCB (unwashed) were mixed evenly according to the mass ratio of 10/1, which was the same mass ratio for mixing HB (unwashed) and sand in the HB-Column. Sand-Column was filled only with sand as a control group. The mixed fillers were filled into the columns with hierarchical compaction method. Washed and dried gravels were placed on the top of columns to prevent current scour.

DW was pumped into the three columns by peristaltic pumps at a rate of 15 mL/min for 3 h. AS was pumped at the same rate for 3 h after the DW was pumped for 3 h. AS included 1 mg/L NO_3_^−^, 3 mg/L NH_4_^+^, 120 mg/L CaCl_2_ and 3 mg/L PO_4_^3−^. The inflow velocity was calculated according to the rainfall intensity formula, Equation (4), in Nanjing, China. The catchment ratio of bioretention facilities was set to 15.

(4) $$i=\frac{64.3+53.8\mathrm{lg}P}{{\left(t+32.9\right)}^{1.011}},$$

where, *i* is the rainfall intensity (mm); *P* is the rainfall probability; *t* is the lasting time.

Furthermore, 50 mL effluent was collected every 20 min by the effluent tubing at the bottom to detect the ammonium contents. The detection methods were the same as above.

## 3. Results and Discussion

### 3.1. Physicochemical Properties of Additive Fillers

Physicochemical properties of four additive fillers are demonstrated in Table 1. As an additive filler in bioretention facilities, PCB had outstanding physical properties in stormwater treatment compared to other materials for its lightness, porosity and hydraulic performance. It had the smallest natural bulk densities (*ρ*), the highest pore ratio (*e*) and BET as large particle materials, the highest saturated moisture content (*ω_sat_*), and the highest permeability coefficient (*K*). These characterizations bring PCB the excellent water-holding capacity and the efficient hydraulic conductivity to minimize overland flooding in biorention systems.

As for chemical properties, PCB is acidic as a certain amount of carbamate has been formed during the curing process of PCB, and the amino acids dissolved in water would lead to acidity in the suspension of PCB. CEC was 37.5 cmol/kg for PCB, far higher than other materials. The values of CEC are related to the quantities of surface acidic functional groups, and it is positively associated with ammonium adsorption capacity. The CEC of PCB was five times that of HB, because of hydroxyl, carboxyl and other acidic functional groups contained in polyurethane materials, resulting in dissociation producing hydrogen ions forming negative charges when in contact with water molecules, which will improve the cation exchange capacity. The total nitrogen content (TN) of PCB was 2.88%, which was significantly higher than that of HB (0.07%). The increment of TN is predictable, since polyurethane contained urethane which would lead to an increase in TN.

**Figure 2:**
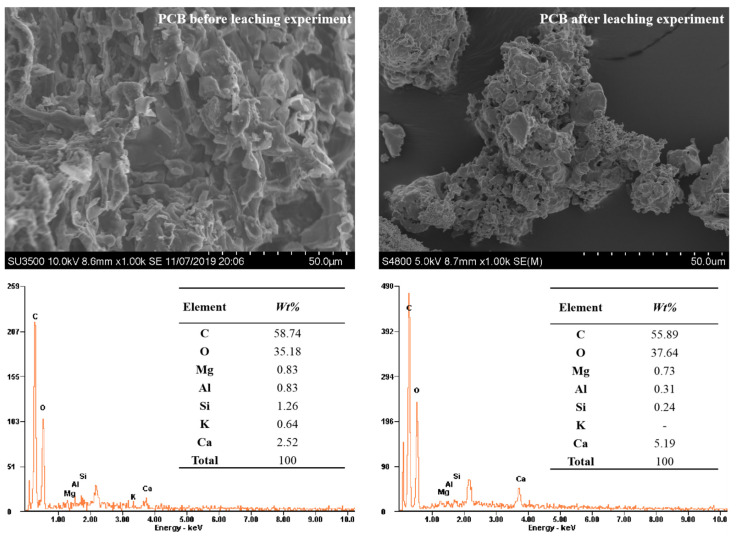
The scanning electron microscopy (SEM) and energy dispersive spectroscopy (EDS) results of polyurethane–biochar crosslinked material (PCB) before and after leaching experiments.

The SEM images and EDS results of original and DW leached PCB are shown in Figure 2. The PCB surface had layered and striated burrs, and inside there were irregular throats with a diameter of about 0.1 mm where the stormwater could flow. After rinsing, the surface of the PCB became smoother, and the hydraulic power of the oscillator rounded off the burrs of PCB. The EDS results showed the change of elements on the surface of PCB before and after leaching. The contents of metal elements (Mg, Al, K) decreased during leaching, which had been washed off the surface of PCB.

### 3.2. Thermal Characterizations of Polyurethane–Biochar Crosslinked Material (PCB)

**Figure 3:**
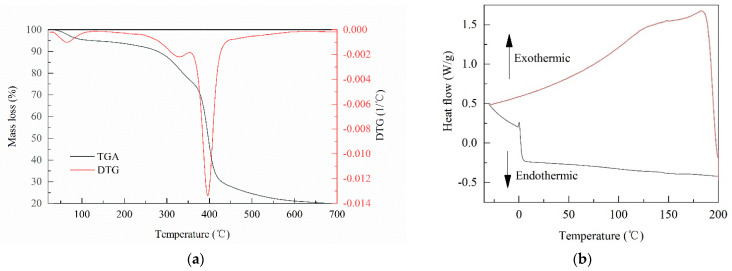
Thermal characterizations of PCB. (**a**) Thermogravimetric analysis (TGA) and differential thermogravimetric analysis (DTG) ramped profiles of the PCB**;** and (**b**) differential scanning calorimetry (DSC) thermograms of the PCB.

In order to evaluate the stability of PCB at different temperatures, two thermal characterizations tests were conducted, measuring the relationship between the physical properties of PCB and temperature. Figure 3 displays the TGA and the DSC testing results of PCB. The TGA data demonstrate that the mass loss process of PCB can be roughly divided into three stages: the first stage was in the range of 28–131 °C, and the mass loss rate was about 5.1%; the second and third stages had faster speeds of mass loss, which were in the range of 262–354 °C and 354–450 °C, respectively. In the whole process of thermogravimetric analysis, PCB was reduced to 19.5% of the initial mass in the end. In the first stage, the mass loss of PCB mainly came from adsorbed water and crystal water in PCB. When the temperature rose to about 262 °C, PCB entered the stage of rapid mass loss. The second and third stages were the main stages of mass loss for PCB. The thermal decomposition of PCB mainly went through the hard segment thermal decomposition and the soft segment thermal decomposition in the second and third stages. The hard segment decomposition temperature of PCB was 262 °C, and the soft segment decomposition temperature was 354 °C. The HB was produced by pine at a 600–700 °C pyrolysis temperature. Hence, it is inferred that the residue of PCB in TGA was HB.

DSC was used to test the crystallization–thermofuse behavior of PCB during the temperature cycle. In the cooling process (200 to –30 °C), there was no crystallization peak. In the heating process (–30 to 200 °C), there was a small melting peak at about 155 °C. Meanwhile, the step peak of the glass transition temperature and cold crystallization peak were not found during heating. According to the TGA and DSC results, it could be concluded that PCB had stable thermal characterizations.

### 3.3. Ammonia Nitrogen Leaching

**Figure 4:**
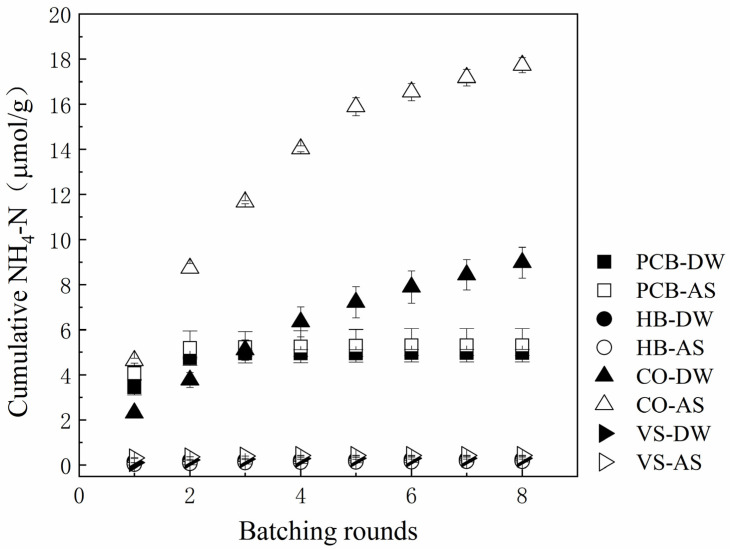
Cumulative ammonia nitrogen (NH_4_-N) leached from additive fillers (PCB, hardwood biochar (HB), compost (CO), and volcanic stone (VS)) in deionized water (DW) or artificial stormwater (AS).

The leaching experiments were conducted to assess the potential leaching quantities of ammonium under working conditions as filler additives. Ammonia nitrogen leaching quantities of additive fillers in DW or AS are enumerated in Table 2. Figure 4 shows the cumulative NH_4_-N leached from materials in DW or AS.

4.98–5.31 μmol/g NH_4_-N were released totally by PCB during the eight rounds of DW and AS leaching experiments. The source of NH_4_-N from PCB may be carbamate produced by polyurethane prepolymer during the foaming and curing process, when the bonds of amino acid were broken due to hydraulic shaking, and NH_4_-N dissolved in DW and AS, which was also the reason for the acidity of the PCB suspension [28]. The first leaching quantities of NH_4_-N from PCB were 3.45–4.07 μmol/g, accounting for 69.25–76.66% of the total rounds, indicating that ammonia nitrogen leaching from PCB mainly occurred in the first round. By the third round of leaching, the contents of NH_4_-N in the leaching solution of PCB-DW and PCB-AS were already lower than 0.01 mg/L. As for the polyurethane material, the shorter the hardening duration time and the longer the leaching immersion period it has in water, the more the types and quantities of nitrogen leached, while the immersion water matrices also have a slight influence on ammonia nitrogen leaching [29]. AS promoted the NH_4_-N leaching quantities and speed of PCB. As coating or crosslinking materials, polyurethane polymers mixed with fertilizers (such as urea) could implement internal nitrogen slow release at a constant speed and increase the release longevity of fertilizers [28].

HB and vs. had lower NH_4_-N leaching quantities. Stable pyrogenic heterocyclic compounds were formed by nitrogen in biochar production under high temperature pyrolysis with higher stability and lower leaching quantities [30]. After weathering and deposition, soluble nitrogen of vs. turned into stable compounds, and this process reduced possibility of the nutrients leaching.

Although the ammonia nitrogen leaching quantities of PCB was higher than that of HB and VS, compared to the compost, humus, manure, coconut and other nitrogen-rich additive materials in stormwater treatment, PCB could be considered as a cleaning additive filler. CO leached 8.98 μmol/g NH_4_-N in DW, and 17.73 μmol/g in AS. Researchers also observed that manure leached 8.85–38.61 μmol/g of NH_4_-N in 105 days of leaching experiments [31], and coconut leached 111.06 mg/g NH_4_-N into the bioretention media in the stormwater simulation [32].

### 3.4. Ammonium Isothermal Adsorption

**Figure 5:**
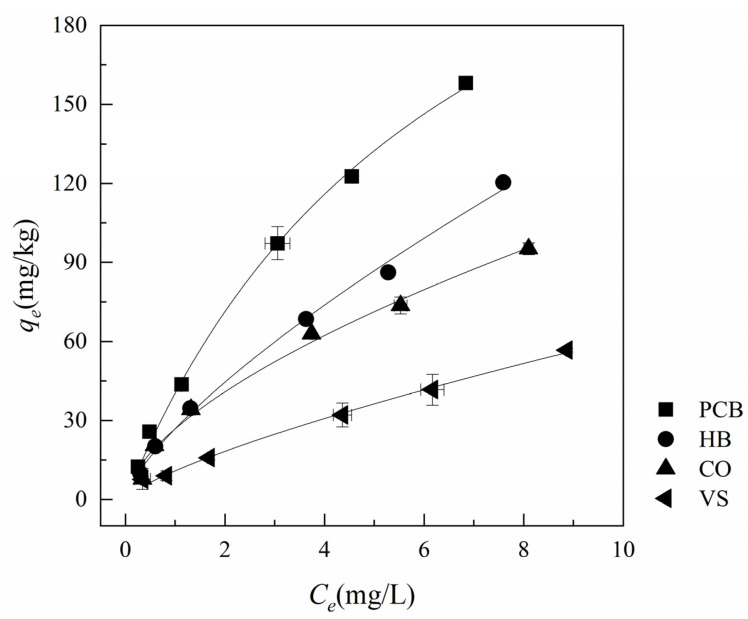
Ammonium adsorption isotherms of DW rinsed additive fillers (PCB, HB, CO and VS).

The isothermal adsorption test results of NH_4_^+^ by four additive fillers after DW leaching are shown in Figure 5. The adsorption capacity of PCB to NH_4_^+^ is significantly higher than that of other materials. Compared with the raw material HB, the equilibrium adsorption capacity of PCB to NH_4_^+^ is 1.23–1.42 times that of HB. The NH_4_^+^ adsorption rates of PCB at different concentrations ranged from 32 to 52%, while the adsorption rates of HB were 24–40%.

**Figure 6:**
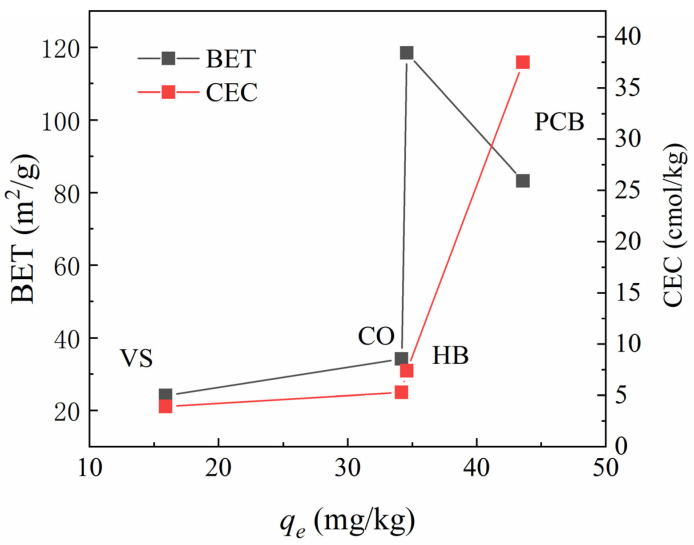
Correlation of ammonium equilibrium in typical runoff concentration with BET and CEC of leached materials.

According to the analysis of physicochemical properties of the materials (Table 1) and comparising with other additive geomedia in stormwater treatment, high BET and CEC were observed to be the main reasons for the NH_4_^+^ adsorption advantage of PCB. In order to explore the decisive factors and influence of ammonium adsorption, a double-factors analysis chart was created to evaluate the relationship between BET and CEC on the performance of ammonium adsorption. Figure 6 shows the relationship between the equilibrium adsorption capacity of materials and BET and CEC under the typical concentration of NH_4_^+^ in typical stormwater runoff (2 mg/L). The high pyrolysis temperature gave rise to the high BET of HB, but its adsorption capacity is weaker than PCB due to the lower CEC. Thus, it can be inferred that the adsorption capacity of NH_4_^+^ by the materials was mainly dependent on CEC. Gai also believed that CEC was the main factor influencing adsorption through adsorption studies of 12 types of biochar [33]. BET also had a slight influence on adsorption, and the larger specific surface area will promote the adsorption reaction, especially for CO. More interestingly, Table 1 illustrated that HB had a higher BET, while crosslinking with polyurethane, the BET of PCB decreased. In essence, the crosslinked polyurethane material formed a honeycomb structure, which can help to absorb more nitrogen and other substances. Li’s study also believed that CEC and morphological characteristics (such as porosity and specific surface area) were the main factors affecting the adsorption of NH_4_^+^ by biochar [34]. As for polyurethane materials, Moawed considered that the adsorption of materials mainly depended on its functional groups on the surface, such as ether group. The ratio of mesopores and microholes in the polyurethane materials remained within a certain range, and it would have a good adsorption performance [35]. Hence, it can be considered that the adsorption of NH_4_^+^ by PCB is mainly dependent on ion exchange, and partly by physical absorption.

To explore the mechanism of NH_4_^+^ adsorption, the results of isothermal adsorption of materials were also fitted with two adsorption models, as shown in Table 3. R^2^ in Table 3 shows that both the Freundlich model and the Langmuir model could fit the NH_4_^+^ adsorption of the four additive fillers well, but the Freundlich model had a higher matching degree, and it was more suitable for simulating the adsorption of polyurethane and biochar materials, which was consistent with the research conclusions of Ahmed [27] and Yao [36]. In the Freundlich models of the four additive fillers, the inverses of characteristic constants (1/*n*) were all less than 1, and the Langmuir model coefficients *R_L_* were between 0 and 1. The two parameters proved that the adsorption of NH_4_^+^ easily occured. The adsorption of NH_4_^+^ by PCB and HB was nonlinear. With the increase of concentration in the solution, its adsorption capacity gradually becomes saturated, which was also confirmed by the bending of the fitting curves in Figure 5. *K_F_* (the Freundlich model’s volumetric affinity parameter), to some extent, proved that compared with HB, PCB had a better adsorption affinity for ammonium. The *q_max_* in the Langmuir model reflected the potential maximum adsorption capacity of the materials. Therefore, PCB can be used as an additive filler with high adsorption performance in stormwater treatment.

### 3.5. Stormwater Infiltration Experiments

Based on the leaching and adsorption experiments, PCB and HB were chosen to verify the actual operational efficiency as additive fillers in bioretention facilities. Column experiments were conducted to simulate the infiltration process of stormwater runoff, evaluate the suitability and feasibility of PCB and make a comparison with the previous single material leaching experiments.

**Figure 7:**
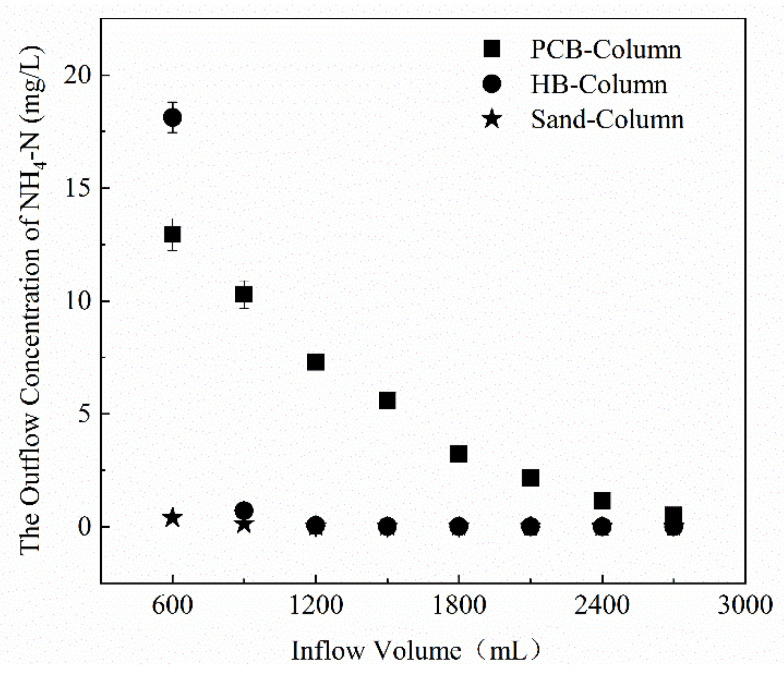
Concentration of NH_4_^+^ in effluent from the PCB-Column, HB-Column and Sand-Column.

The study conducted two rainfall simulations that lasted for 3 h and came once a year. In the first rainfall event, DW was pumped into 3 columns at a rate of 15 mL/min, and NH_4_^+^ in the effluent was detected. Figure 7 shows the changes of NH_4_^+^ concentration in effluent from the columns during the first rainfall simulation. With the addition of PCB, the PCB-Column gradually released NH_4_^+^. After pumping for 40 min, filtrate flowed out from the bottom of the column. The first 50 mL of effluent from the collection had an ammonium content of 12.94 mg/L. As the DW was continuously pumped in, the concentration of NH_4_^+^ gradually decreased to nearly 0. However, the release of NH_4_^+^ in HB-Column was mainly concentrated in the first 50 mL of effluent of the rainfall process, which was much higher than the concentration of effluent in the later stage, fromm 18.12 mg/L it sharply decreased to nearly 0.

To evaluate whether the previous leaching experiments could be used to estimate the stormwater infiltration effluent, the estimated (calculated in leaching experiments) and detected concentrations (calculated in column experiments) are listed in Table 4 for comparison. The estimated concentrations were based on the assumption that the PCB and HB in the columns would release the same quantities of ammonium as in the leaching experiments. Furthermore, 626.73 g of PCB and 834.94 g of HB were added to the two columns, respectively. According to the assumption of the same leaching quantities, it was assumed that after 40 min of pumping in DW, the first 50 mL of effluent NH_4_^+^ concentrations of the two columns should be 50.45 and 3.12 mg/L. However, in reality, the NH_4_^+^ concentration of the PCB-Column was far lower than the estimated value, and so was the total leaching amount. The HB-Column showed abnormalities, and the detected concentration of NH_4_^+^ was 3.8–5.81 times the estimated value. It is predictable that the detected concentration and the total release quantities of NH_4_^+^ were lower than the estimated. During the infiltration process, it had smaller contact surface and less time between filters and DW than in the leaching experiments [37]. What is more, the hydraulic power of the leaching experiments was stronger than that of the infiltration process, which led to the insufficient dissolution and low dissolution rate of the ammonium, and ulteriorly the detected value would be lower than the estimated value. The abnormality of the HB-Column may be caused by the negative charges on the surface of the biochar. The charges promoted the leaching of NH_4_^+^ which should have adhered to the surface of the additive fillers and was easily washed away [38]. In general, the results of the leaching experiments would overestimate the quantities of ammonium leaching and could be considered as a limiting factor for long-term operation [39].

**Figure 8:**
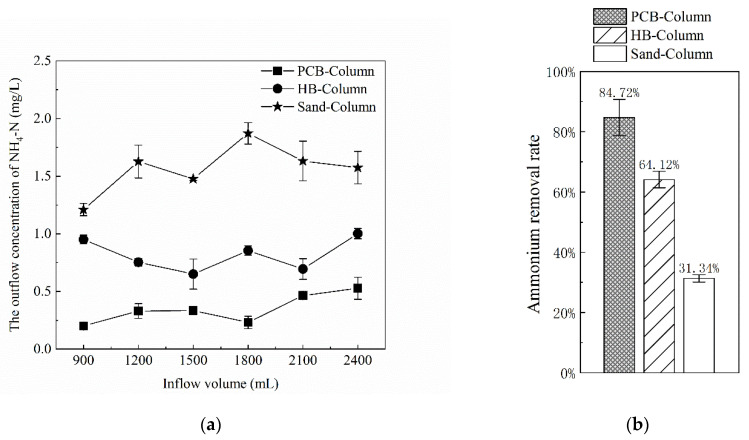
Concentration and mean removal rates of NH_4_^+^ in outflows from the three columns in the second simulated rainfall event. (**a**) The outflow concentrations of NH_4_^+^ from the three columns; and (**b**) the mean removal rates of NH_4_^+^ from the three columns.

In the second rainfall event, DW was changed to AS to be pumped in, and other testing conditions remained. Considering the influence of the first rainfall simulation, the study analyzed the effluent concentration after AS had been pumped for 1 h. The effluent concentration of NH_4_^+^ in the PCB-Column and the HB-Column fluctuated somewhat, but remained within a certain range. The mean values of NH_4_^+^ removal rates were computed using effluent concentration data and are shown in Figure 8 for the three columns.

The average NH_4_^+^ removal rate of the PCB-Column was 84.72%, which was significantly higher than that of the HB-Column (64.12%) and the Sand-Column (31.34%). In the isothermal adsorption experiments, the adsorption rates of PCB and HB to NH_4_^+^ at 2 mg/L were 43.6% and 34.6%, respectively, which were consistent with the results of the column experiments. According to the prediction of *q*_max_ from the Langmuir model in the adsorption experiments, the PCB-Column and the HB-Column would lose the adsorption capacity of NH_4_^+^ when the inflow volume reached 193.39 L and 233.97 L, respectively. After mixing with sand as filler in columns, PCB and HB were greater influencing factors on NH_4_^+^ adsorption: shorter contact time, reduced contact surface and reduced hydraulic power, all of which would affect the working effects of additive fillers in stormwater treatment [40]. Hydrophilicity is an important factor affecting the effect of ammonium removal in the infiltration process of materials. Absorption efficiency would get worse when the additive geomedia could not have full access to the runoff due to the poor hydrophilicity. As a polyurethane material, PCB has strong hydrophilicity due to its concave and convex surface, so its adsorption capacity was stronger than HB.

The column experiments simulated the operation efficiency of the bioretention facilities with the addition of PCB or HB in the filtration layer. In general, the addition of PCB and HB would improve the adsorption of NH_4_^+^ in the facilities. Under the once-a-year rainfall intensity and only considering the role of geomedia, the PCB modified filtration layer could absorb most of the NH_4_^+^ from the 15 site areas. In the long run, the ammonium adsorption capacity of the additive fillers will be gradually saturated, losing purification capacity. However, with the dry-wet cycle process and the joint action of vegetation and root system, it will help to restore the adsorption capacity of the additive fillers [41], however, the quantitative longevity needs further research.

## 4. Conclusions

Additive fillers in the filtration layers are the key to improve the capacity of pollutants removal and permeability in bioretention systems. The physicochemical properties of PCB and other compared materials were studied and discussed. After modification with polyurethane, the PCB became acidic, and the saturated moisture content was increased by 1.96 times that of HB. The permeability coefficient was increased by 1.3 × 10^2^ times, as well as the CEC was increased by 5.06 times. Through TGA and DSC tests, the stable thermal characterizations of PCB had been confirmed.

From the perspective of ammonia nitrogen release and ammonium adsorption, PCB is a feasible additive filler in stormwater treatment. PCB leached 4.98–5.31 μmol/g NH_4_-N in batching experiments, which were produced in the curing process of polyurethane foam. In the isothermal adsorption experiments, PCB showed the best adsorption, with 32–52% adsorption capacity of NH_4_^+^.

The column experiment results were compared with the leaching tests, and it was found that the NH_4_-N leaching concentrations from the column tests were lower than the predicted value calculated by the leaching test results due to the short contact time and lighter hydraulic power. In addition, column experiments were conducted to simulate the adsorption efficiency of the additive fillers in actual operation, which confirmed that the PCB had a good adsorption capacity of NH_4_^+^ in runoff.

In general, PCB had high BET, CEC and ion exchange capacity, and it is a suitable additive filler in stormwater treatment, which can improve water retention, reduce the leaching of ammonia nitrogen and improve water quality. At the same time, in order to avoid the first use of excess ammonia nitrogen concentration of effluent, it can be used after several cycles of DW washing. However how to further improve the removal effect of nitrate and other pollutants and the longevity of the material needs further study.

## Figures and Tables

**Table 1:** Physicochemical properties of additive fillers.

Media Material	*ρ*^1^ (g/cm^3^)	*e* ^2^	Particle Size (mm)	*ω_sat_* (%) ^3^	*K* (cm/s) ^4^	BET (m^2^/g)	pH	CEC (cmol/kg)	TN ^5^ (%)
Polyurethane–biochar crosslinked material (PCB)	0.165	3.20	1–2	383.50	8.56 × 10^−2^	83.14	6.62	37.5	2.88
Hardwood biochar (HB)	0.378	3.88	<0.5	195.65	6.57 × 10^−4^	118.45	8.80	7.4	0.07
Compost (CO)	0.314	1.66	3–5	267.61	7.62 × 10^−2^	34.10	7.08	5.3	1.25
Volcanic stone (VS)	0.828	0.59	3–5	22.43	7.21 × 10^−1^	24.05	6.78	3.9	0.03

^1^ *ρ* is natural bulk density. ^2^ *e* is pore ratio. ^3^ *ω_sat_* is saturated moisture content. ^4^ *K* is permeability coefficient. ^5^ TN is total nitrogen.

**Table 2:** Ammonium leaching quantities of filler materials in deionized water (DW) or artificial stormwater (AS).

Material	8 Rounds (μmol/g)	1st Round (μmol/g)	1st Round/8-Rounds
PCB-DW	4.98	3.45	69.25%
PCB-AS	5.31	4.07	76.66%
HB-DW	0.25	0.16	62.50%
HB-AS	0.17	0.05	29.31%
CO-DW	8.98	2.30	25.66%
CO-AS	17.73	4.63	26.10%
VS-DW	0.30	0.10	33.33%
VS-AS	0.41	0.31	75.17%

**Table 3:** Parameters for Freundlich and Langmuir isotherms of ammonium adsorption.

Material	Freundlich			Langmuir			
	*K_F_*	1/*n*	R^2^	*q*_max_ (mg/kg)	*K_L_*	R^2^	*R_L_*
PCB	85.125	0.691	0.996	617.149	0.150	0.995	0.399–0.930
HB	53.689	0.729	0.996	560.439	0.093	0.984	0.518–0.956
CO	26.795	0.608	0.986	142.992	0.219	0.986	0.314–0.901
VS	10.865	0.750	0.994	152.247	0.064	0.983	0.608–0.969

**Table 4:** Comparison of predicted and detected concentration and total release of ammonium in column experiments.

Columns	Concentration of the First 50 mL of Effluent (mg/L)		Total Leaching Quantities (mg)	
	Predicted	Detected	Predicted	Detected
PCB-Column	50.45	12.94	43.70	16.82
HB-Column	3.12	18.12	2.92	11.11

## Data Availability

The data presented in this study are available in this article.
